# Integrating Digital Health Solutions with Immunization Strategies: Improving Immunization Coverage and Monitoring in the Post-COVID-19 Era

**DOI:** 10.3390/vaccines12080847

**Published:** 2024-07-28

**Authors:** Grazia Pavia, Francesco Branda, Alessandra Ciccozzi, Chiara Romano, Chiara Locci, Ilenia Azzena, Noemi Pascale, Nadia Marascio, Angela Quirino, Giovanni Matera, Marta Giovanetti, Marco Casu, Daria Sanna, Giancarlo Ceccarelli, Massimo Ciccozzi, Fabio Scarpa

**Affiliations:** 1 Unit of Clinical Microbiology, Department of Health Sciences, “Magna Græcia” University of Catanzaro—“Renato Dulbecco” Teaching Hospital, 88100 Catanzaro, Italy; graziapavia@unicz.it (G.P.); nmarascio@unicz.it (N.M.); quirino@unicz.it (A.Q.); mmatera@unicz.it (G.M.); 2 Unit of Medical Statistics and Molecular Epidemiology, Università Campus Bio-Medico di Roma, 00128 Rome, Italy; chiara.romano@unicampus.it (C.R.); m.ciccozzi@unicampus.it (M.C.); 3 Department of Biomedical Sciences, University of Sassari, 07100 Sassari, Italy; aciccozzi@uniss.it (A.C.); c.locci3@phd.uniss.it (C.L.); darsanna@uniss.it (D.S.); fscarpa@uniss.it (F.S.); 4 Department of Veterinary Medicine, University of Sassari, 07100 Sassari, Italy; iazzena@uniss.it (I.A.); pascalenoemi3@gmail.com (N.P.); marcasu@uniss.it (M.C.); 5 Department of Sciences and Technologies for Sustainable Development and One Health, Università Campus Bio-Medico di Roma, 00128 Rome, Italy; giovanetti.marta@gmail.com; 6 Instituto René Rachou, Fundação Oswaldo Cruz, Belo Horizonte 30190-002, Minas Gerais, Brazil; 7 Climate Amplified Diseases and Epidemics (CLIMADE), Brasilia 70070-130, Goias, Brazil; 8 Department of Public Health and Infectious Diseases, University Hospital Policlinico Umberto I, Sapienza University of Rome, 00161 Rome, Italy; giancarlo.ceccarelli@uniroma1.it

**Keywords:** digital health tools, immunization strategies, vaccination practice, digital health interventions

## Abstract

The COVID-19 pandemic underscored the critical importance of vaccination to global health security and highlighted the potential of digital health solutions to improve immunization strategies. This article explores integrating digital health technologies with immunization programs to improve coverage, monitoring, and public health outcomes. It examines the current landscape of digital tools used in immunization initiatives, such as mobile health apps, electronic health records, and data analytics platforms. Case studies from different regions demonstrate the effectiveness of these technologies in addressing challenges such as vaccine hesitancy, logistics, and real-time monitoring of vaccine distribution and adverse events. The paper also examines ethical considerations, data privacy issues, and the need for a robust digital infrastructure to support these innovations. By analyzing the successes and limitations of digital health interventions in immunization campaigns during and after the COVID-19 pandemic, we provide recommendations for future integration strategies to ensure resilient and responsive immunization systems. This research aims to guide policymakers, health professionals, and technologists in leveraging digital health to strengthen immunization efforts and prepare for future public health emergencies.

## 1. Introduction

Immunization has been long recognized as one of the main effective public health interventions, acting as the core of the primary healthcare system [1,2]. Advances in vaccine research significantly reduced the burden of infectious diseases, preventing millions of deaths each year [1,3,4]. On 25 May 2024, a landmark study conducted by the World Health Organization (WHO) [5] revealed that global immunization efforts, over the past 50 years, have preserved approximately 154 million people, with an average of six lives saved every minute [5]. Specifically, vaccination against 14 diseases, including diphtheria, measles, pertussis, and polio, has reduced infant mortality by 40% globally and more than 50% in Africa [5,6]. Among these, the measles vaccine had the greatest impact on public health, accounting for 60% of lives saved, and is expected to remain a leading contributor to reducing mortality [6,7]. The eradication of smallpox [8], the near-elimination of polio [9], as well as the recent advancements targeting diseases like malaria [10] and cervical cancer [11], further emphasize the transformative power of immunization. The COVID-19 pandemic has brought unprecedented challenges to global health, underscoring the essential role of immunization in safeguarding public health and security [12,13,14,15]. To date, 5.47 billion doses of COVID-19 vaccines have been administered worldwide, with over 56% of the population receiving a complete primary series [12]. In EU/EEA countries, according to the latest report from the European Centre for Disease Prevention and Control (ECDC), a total of 981 million COVID-19 vaccine doses (including first, second, and booster doses) have been administered. Approximately 30 million doses were provided in 2021, with a decrease to around 199,000 doses by May 2023 [16]. However, the efforts deployed to provide wide COVID-19 immunization coverage strained health systems in 2020 and 2021. It resulted in dramatic setbacks to routine immunization services that, in turn, caused reductions in community vaccine coverage with a substantially increased risk of disease [17]. The global resurgence of measles outbreaks [18], the interruption of the polio eradication program [19], and increasing reports of diseases such as diphtheria and pertussis, have been primarily attributed to the decline in vaccine coverage caused by the disruptions during the COVID-19 pandemic [20,21,22]. According to the WHO/UNICEF immunization data portal, vaccination coverage for diphtheria, tetanus toxoid, and pertussis (DTP), as well as poliomyelitis, decreased from 86% in 2019 to 82% in 2020–2021 pandemic years [23]. In this regard, the Immunization Agenda 2030 (IA2030), ratified by all member states in 2020, commits to halving the number of zero-dose children worldwide, ensuring children who missed immunizations are caught up, and restoring vaccination services to pre-pandemic levels [24]. In the post-COVID-19 era, digital health solutions, such as electronic health records (EHRs), mobile health applications (mHealth), telemedicine, and other smart devices with Internet connections, could become the future choices for enhancing immunization strategies and rebalancing global vaccination coverage [25]. These devices allow the gathering of an individual’s healthcare information at the patient’s home, improving vaccine distribution logistics, monitoring adverse events more effectively, and ensuring comprehensive immunization coverage, particularly in underserved regions [25]. This review article examines the successes and limitations of digital health interventions in immunization campaigns during and after the COVID-19 pandemic. It provides an overview of future integration strategies aimed at creating responsive and resilient immunization systems. The research is designed to guide policymakers, health professionals, and technologists in leveraging digital health solutions to strengthen immunization efforts and better prepare for future public health emergencies.

## 2. Digital Health Tools in Immunization

The digital revolution has significantly transformed the development of products and services across various domains, including human health [26,27]. The COVID-19 pandemic acted as a practical proving ground for the application of new digital health solutions [28], highlighting their potential use in the post-pandemic landscape. Indeed, their strategic adoption could be essential to restore and enhance global immunization rates in the post-pandemic era, ensuring that no population is left behind in the effort to prevent vaccine-preventable diseases [29]. In this regard, a wide range of digital tools that facilitate accessibility, efficiency, and effectiveness across healthcare systems toward immunization programs was implemented. The characteristics, advantages, and disadvantages of each digital health solution to improve immunization strategies are summarized in Table 1.

A source of real-world data is represented by the EHRs, a centralized platform for comprehensive and longitudinal storing and managing of patient records, including tracking of vaccination histories and identification of individuals due for immunization [30]. Recent advances in EHRs technology, such as interoperability standards and integration with public health databases, have enabled seamless data sharing across healthcare systems, facilitating coordinated efforts in monitoring vaccine coverage and distribution [31,32]. EHRs also support automated reminder systems and clinical decision support tools, which can prompt healthcare providers to administer vaccines in a timely way and alert patients about upcoming or missed immunizations [33]. However, the adoption of EHRs is not without challenges. Issues such as data privacy concerns [34], the high cost of implementation, and the need for significant training for healthcare professionals can delay their widespread use [35,36]. Indeed, with the increased use of EHRs, a large volume of data is becoming increasingly available and accessible to both authorized and unauthorized users, which can impair the confidentiality and privacy of patients’ personal information [35,36]. Additionally, the variability in EHRs system capabilities and the lack of universal standards can result in inconsistencies in data quality and accessibility [37]. Despite these disadvantages, the strategic use of EHRs in immunization programs could lead to improved public health outcomes by ensuring comprehensive vaccination coverage and facilitating rapid response to emerging infectious threats.

During the COVID-19 pandemic, global users increased their time spent using mobile apps across various fields, including healthcare applications [38]. Among these, mHealth has emerged as a powerful tool to communicate with clinicians and maintain routine immunization schedules, also in rural areas [39,40,41]. These applications offer several distinctive options, including real-time access to vaccination records, personalized immunization schedules, and automated reminders for upcoming or overdue vaccines [39,41,42]. The advances in mHealth technology, such as integration with EHRs, have further amplified their potential application in healthcare settings [43]. Another useful utilization of mHealth apps concerns promoting immunization uptake and education among diverse populations [39,41,44]. Indeed, the success of vaccination programs depends not only on the equitable availability of safe vaccines but also on their acceptance and uptake by the population. In this regard, apps also serve as platforms for healthcare providers to disseminate real-time updates during immunization campaigns, such as vaccine availability and local clinic hours to ensure comprehensive vaccine coverage [29]. Privacy concerns, the digital gap affecting access to technology, and the need for rigorous validation of app efficacy can pose significant barriers regarding their utilization [45]. Despite these disadvantages, mHealth applications hold substantial promise for improving immunization rates by providing convenient, user-friendly platforms for vaccine management and education, thereby supporting public health initiatives and resilience against future pandemics. During the COVID-19 pandemic, virtual communication platforms facilitated remote consultations between healthcare professionals and patients. Also, they enabled the creation of management dashboards to streamline workflows, optimize resource allocation, and focus on patient immunization strategies [46]. The transition to telemedicine and telehealth platforms was significantly strengthened and accelerated during the COVID-19 pandemic. Such features affected not only COVID-19 patients but also all individuals interacting with the healthcare system [47]. This change led to a significant decrease in in-person patient visits, coupled with a substantial rise in telehealth consultations [48]. Although telemedicine and telehealth service utilization have presently decreased [49], their application in the post-pandemic era appears to overcome geographical barriers and improve access to immunization services worldwide. These platforms could enable healthcare providers to conduct virtual consultations, assess vaccination needs, and monitor adverse reactions remotely [50]. In regions with limited access to healthcare and in-person patient visits, telemedicine could facilitate timely interventions and follow-up care, ensuring that populations receive essential vaccines without requiring physical visits to clinics [51]. However, the widespread adoption of telemedicine and telehealth is impaired by challenges including data privacy concerns, the digital gap affecting access in underserved areas, and the need for substantial investment in infrastructure and training [52].

During the pandemic, surveillance systems, such as the COVID-19 Open Research Database, have facilitated rapid data exchange among researchers and the consultation of vast amounts of genomic insights on the global circulation of SARS-CoV-2 variants [53]. Similarly, data analytics and surveillance systems could be a key chance to ensure both national and global health security, such as the optimization of immunization programs on a global scale [54]. These systems leverage big data to track vaccination coverage rates, identify high-risk populations, such as patients with chronic or persistent infection [14,55,56], and predict disease outbreaks based on poor immunization coverage [57]. Moreover, surveillance systems facilitate early detection of vaccine-preventable diseases and adverse events following immunization (AEFIs), enabling swift responses and containment measures [58]. However, ensuring data accuracy, privacy protection, and interoperability across healthcare systems all remain ongoing challenges that require collaborative efforts among stakeholders to strengthen global immunization efforts and achieve health equity [59,60]. Artificial intelligence (AI) and machine learning (ML), during the COVID-19 pandemic, were an essential tool for the rapid analysis of immunization coverage by analyzing wide datasets to optimize vaccine distribution, predict disease outbreaks, and personalize healthcare interventions [61]. In particular, AI platforms generated data summaries from various sources, enabling real-time monitoring of vaccination progress in high-risk populations [61,62]. Also, ML models have enhanced vaccine efficacy studies by identifying patterns in vaccine responses and adverse events, informing regulatory decisions and vaccine development efforts [63]. AI-driven analytics are, also, capable of personalizing vaccine outreach by tailoring communication strategies to specific demographic groups, thereby addressing vaccine hesitancy more effectively [64]. However, ensuring AI ethics, data privacy, and algorithm transparency are critical considerations in deploying these technologies within immunization programs [65]. Collaborative efforts among researchers, policymakers, and healthcare providers are essential to harness AI’s full potential in enhancing global immunization coverage and public health outcomes.

The healthcare industry is being shaped by the Internet of Things (IoT) revolution characterized by technological advancements, increased economic efficiency, and significant social benefits [66,67]. IoT devices, in the last few years, have played a crucial role in monitoring vaccine storage conditions, ensuring vaccine potency, and optimizing supply chain logistics [68]. IoT sensors track temperature, humidity, and other environmental factors in storage facilities, providing real-time alerts and notifications to healthcare providers if conditions deviate from optimal ranges [69]. Furthermore, IoT-enabled inventory management systems streamline vaccine distribution by automating stock replenishment, minimizing stockouts, and improving supply chain efficiency [70]. As healthcare systems continue to adopt IoT solutions, ensuring data security, interoperability, and scalability are essential to maximize their impact on global immunization efforts and improve healthcare delivery worldwide [71].

**Table 1:** Digital health solutions: characteristics, advantages, and disadvantages to improve immunization strategies.

Digital Health Solution	Characteristics	Advantages	Disadvantages	References
Electronic Health Records (EHRs)	Digitalization of medical records, real-time updates and access	Improved vaccination tracking, quick access to patient information, reduction in errors	Requires significant investment in infrastructure and training, data privacy and security concerns	[30,31,32,33,34,35,36,37]
Mobile Health Applications (mHealth)	Smartphone apps for vaccination reminders, health information, and monitoring	Accessibility, ease of use, increased patient engagement, reminders for vaccinations	Limited access in areas with poor connectivity, dependence on digital literacy	[29,39,40,43,45]
Telemedicine and Telehealth Platforms	Remote medical consultations via video calls and messaging	Improved access to healthcare, reduced travel time and costs, continuity of care during outbreaks	Limited by internet access and technology infrastructure, potential challenges with patient–provider communication	[47,49,50,52]
Data Analytics and Surveillance Systems	Analysis of large datasets to identify trends and predict vaccine needs	Better resource allocation, identification of at-risk areas, data-driven decision-making	Requires advanced technical skills, risks related to data quality and privacy	[54,57,58,59]
Artificial Intelligence (AI) and Machine Learning (ML)	Predictive analytics, automation of data processing	Improved predictive capabilities, efficient resource allocation, personalized health interventions	Ethical concerns, potential biases in algorithms, need for large amounts of data for training	[61,62,63,64,65]
Internet of Things (IoT) Devices	Network of interconnected devices for real-time monitoring	Real-time tracking of vaccine storage conditions, improved logistics, immediate reporting of adverse events	Security vulnerabilities, high implementation costs, dependency on reliable network connectivity	[66,69,70,71]

## 3. Case Studies and Applications

Digital connectivity platforms enabled healthcare professionals and patients to engage remotely during the COVID-19 pandemic, while also supporting the development of management dashboards designed to optimize workflows, allocate resources efficiently, and enhance patient care [25]. Nevertheless, their potential efficacy in global immunization strategies, mainly in the post-pandemic era, remains poorly understood, encountering numerous barriers within the healthcare setting. Here, selected case studies are considered to illustrate the application of digital health interventions to strengthen immunization strategies.

### 3.1. Case 1

A machine learning-based framework for processing heterogeneous EHRs was developed [72]. The researchers designed and validated a convolutional autoencoder (ConvAE) to generate informative vector-based representations of millions of patients from various hospital datasets. In particular, an EHR dataset of approximately 4.2 million de-identified patients from the Mount Sinai Health System data warehouse, covering the period from 1980 to 2016, was analyzed. This large and diverse urban hospital in New York, NY, generated substantial volumes of structured, semi-structured, and unstructured data from inpatient, outpatient, and emergency room visits. The EHR dataset included patients’ follow-up data for up to 12 years unless they were transferred or moved outside the hospital’s network. The researchers demonstrated that disease progression, symptom severity, and comorbidities significantly contribute to the phenotypic variability of patient’s complex disorders. For example, type 2 diabetes patients were divided into subgroups based on comorbidities and symptom severity. These findings highlight that ConvAE-derived features provide generalizable insights across different clinical settings, contributing to the development of next-generation clinical systems capable of handling extensive patient records and supporting clinicians in stratifying populations. This approach could be particularly useful in identifying high-risk groups during immunization programs, given the higher risk of adverse clinical outcomes, including more frequent hospitalizations and increased length of stay, registered by at-risk populations compared to the general population. However further refinement and broader application are necessary to address its limitations and enhance its clinical utility. The study’s multi-disease clustering might be influenced by noise and biases inherent in EHR data. Minimal data engineering preserves information but may also introduce hospital-specific biases and redundant data. Moreover, the reliance on Systematized Nomenclature of Medicine Clinical Terms (SNOMED-CT) codes for identifying patient cohorts might include false positives, affecting the accuracy of learned representations. Employing more refined phenotyping algorithms could improve cohort selection to support immunization efforts.

### 3.2. Case 2

To promote health and well-being for individuals of all ages, particularly newborns and children under five years in resource-limited settings, extensive vaccination data generated by low-cost EHRs were provided in Pakistan [73]. This digital approach allowed to improve data-driven actions and decision-making for the management of immunization programs and, also, to enhance immunization coverage and overall public health. Indeed, from January to December 2019, monthly program indicator monitoring increased vaccination from 44% to 88% and improved geographical coverage by 85% in a polio-endemic area, such as Karachi. Analysis of daily average vaccines administered during awareness efforts, compared to routine activities, showed increases of 103%, 154%, and 180% for Pentavalent-3, Measles-1, and Measles-2 vaccines, respectively. These insights provide evidence on how EHRs, by access to big vaccine data, have improved drive efforts and data-driven decisions in high-risk areas. The real-time immunization data generated by low-cost EHRs, based on the Android system, have proven a powerful tool to monitor immunization efforts and to ensure coverage in missed communities through targeted strategies. These results highlight several key directions for future research, particularly the need to integrate technology and data into government operations systematically. Effective health policy development relies on the continuous updating of information. However, transitioning and adapting government procedures to incorporate these data proved to be a lengthy and challenging process. Although the Electronic Immunization Registry was fully implemented across the Province by March 2018, meaningful improvements in the utilization of incoming data by the Government were only observed by March 2019. Future research could explore strategies for training program managers and supervisors, who have relied on paper-based reporting systems for years, to become more responsive and adaptable in real-time scenarios.

### 3.3. Case 3

In Bangladesh, vaccination rates for children living in rural, remote regions and urban street settings continue to be low. To address this issue, an experimental study was conducted over 12 months using an mHealth app designed to improve vaccination coverage for children aged 0–11 months in these regions [40]. The intervention utilized software called “mTika” (accessed at https://innov.afro.who.int/emerging-technological-innovations/mtika-3423 on 20 June 2024) which was integrated into the existing public health system to electronically register births and send vaccination reminders to mothers via text messages. Health assistants, vaccinators, and supervisors in the intervention areas were provided with Android smartphones running mTika, while mothers used their existing cell phones. As a result of the intervention, full vaccination coverage increased significantly—from 58.9% to 76.8% in rural areas and from 40.7% to 57.1% in urban areas. The intervention positively impacted age-appropriate vaccination coverage across all age groups. Qualitative data indicated high acceptance of the intervention among participants. This study demonstrates that an mHealth-based approach can effectively enhance vaccination coverage in rural, hard-to-reach, and urban street-dweller communities in Bangladesh. This successful small-scale initiative could serve as a model for other low-income countries. However, several limitations related to its design and the implementation of the mTika intervention were observed. Due to constraints in time and funding, the research could not include a large cohort of children eligible for full vaccination, which would have required a 12-month intervention and an additional 12-month follow-up period. This limitation impacted the ability to fully assess vaccination coverage over time, as compliance often diminishes due to factors such as aging, relocations, and difficulties in maintaining follow-up. Additionally, the mTika intervention, implemented through the existing public health system, lacked rigorous standardization of procedures, randomization of Pakistan’s immunization centers, or contemporaneous controls. Moreover, a substantial number of parents could not produce immunization cards during surveys, further complicating data accuracy. Implementation challenges included the development of new software, coordination among various stakeholders, and varying levels of health worker capacity. Future developments should focus on enhancing maternal engagement with mTika, following up with registered mothers who did not submit birth notifications, and reaching out to those not enrolled during pregnancy.

### 3.4. Case 4

Acceptance of COVID-19 vaccines has significantly varied worldwide, influenced by vaccine hesitancy due to misinformation. To address this, the WHO-EU collaboration launched the COVID-19 InfoVaccines initiative in February 2021 across six eastern European countries [74]. The program aimed to provide healthcare providers with evidence-based answers to common questions about COVID-19 vaccines, helping individuals make informed decisions. COVID-19 InfoVaccines, available in seven languages and promoted through social media, attracted 262,592 users from 11 February 2021 to 31 January 2023. Users primarily sought information on vaccine efficacy, safety, co-administration, dose intervals, and interchangeability, with interests shifting based on the evolving epidemiological context. Engagement with the platform was substantial, resulting in an average engagement rate of 71.61%. The platform saw access from 231 countries and territories. Due to the vaccine misinformation and its potential to negatively impact health decisions, initiatives like COVID-19 InfoVaccines could play a crucial role in offering accessible and reliable information to encourage immunization. However, the results of the study reveal that most users accessed the site via mobile devices and that older adults exhibited greater engagement with COVID-19 information on Facebook. These insights are crucial for refining communication strategies. These data suggest that while the site effectively reached a broad audience, video content was particularly impactful for older users. This underscores the importance of employing diverse content formats in health communication strategies to improve both engagement and overall effectiveness.

### 3.5. Case 5

Despite childhood vaccination being a crucial public health strategy for reducing child mortality and morbidity, data on basic vaccination coverage among children aged 12 to 23 months in Ethiopia indicated only 43% coverage. To perform prediction of childhood vaccination coverage, this study evaluated different ML algorithms to generate association rules that established immunization coverage among children aged 12–23 months [75]. Using a cross-sectional design with a two-stage sampling method, data from 1617 children were extracted from the Ethiopian Demographic and Health Survey dataset. The PART algorithm was identified as the best-performing ML model, achieving 95.53% accuracy in predicting childhood vaccination. Key predictors of vaccination identified in the study included antenatal care (ANC) visits, institutional delivery, health facility visits, higher maternal education, and wealth status. Seven predictive rules were derived, including one that indicated an 86.73% vaccination probability when wealth status was high, ANC visits were adequate, and residency was urban. These findings highlight the importance of targeted interventions, such as enhancing ANC visits, promoting institutional deliveries, improving maternal education, and addressing economic disparities to improve childhood vaccination rates. Although the ML algorithms are effective for classifying and predicting childhood vaccination patterns, they lack coefficients such as odds and incidence rate ratios. As a result, the strength and direction of the associations between variables remain unspecified.

## 4. Challenges and Recommendations

The digital divide represents a significant obstacle in integrating digital health solutions with immunization strategies, particularly in the post-COVID-19 era. This divide manifests in various ways, notably in access to technology and digital literacy. In many regions, especially low- and middle-income countries and rural areas, there is limited access to essential technology, such as smartphones, computers, and reliable internet connections. This lack of access hinders the ability of individuals and healthcare providers to effectively utilize digital health tools. Additionally, even when technology is available, varying levels of digital literacy can prevent its effective use. Many individuals, particularly older adults and those in underserved communities, may lack the skills needed to navigate digital platforms, apps, or online health resources, leading to lower engagement with digital health solutions [76]. Economic barriers further compound the issue. The cost of technology can be prohibitive for many individuals and healthcare facilities, particularly in developing countries. High costs associated with devices, internet services, and software subscriptions limit the widespread adoption of digital health tools. Geographic disparities also play a significant role. Urban areas typically benefit from better infrastructure and more resources compared to rural areas, resulting in significant differences in the availability and quality of digital health services, which further exacerbates health inequities [77].

To overcome these aspects of the digital divide, targeted interventions are essential. Investing in and improving digital infrastructure, particularly in underserved regions, is crucial. Enhancing digital literacy through educational programs is another key strategy. These programs should target both healthcare providers and the general population, offering training on how to use digital health tools effectively [78]. Community centers, including elderly or geriatric centers, libraries, and schools can serve as venues for digital literacy training. Additionally, developing digital health solutions that are tailored to local contexts and languages can increase usability and adoption. Involving local communities in the design and implementation process ensures that the solutions meet their specific needs and circumstances. By addressing the digital divide through strategic investments and educational initiatives, digital health solutions can reach and benefit a broader population. This approach is essential for improving immunization coverage and monitoring, leading to better public health outcomes and a more robust response to future health challenges in the post-pandemic world. Anyhow, it must be considered that the resistance to change among healthcare professionals and the public poses a significant challenge to the integration of digital health solutions with immunization strategies. This resistance often stems from unfamiliarity with new technologies and skepticism about their efficacy and reliability [79]. Healthcare professionals, accustomed to established methods and workflows, may be hesitant to adopt digital tools that require learning new systems and altering their practices. Concerns about the reliability of digital solutions, potential disruptions to patient care, and the time investment needed to adapt to new technologies can contribute to this reluctance [80]. Moreover, the public may also exhibit resistance due to a lack of understanding or trust in digital health solutions. Misinformation and fears about data privacy and security can lead to skepticism about the benefits and safety of digital tools for health management. This is especially true in populations with low digital literacy or previous negative experiences with technology [81]. To address these challenges, it is essential to implement strategies that promote acceptance and confidence in digital health solutions. Engaging healthcare professionals early in the development and implementation process can help tailor solutions to their needs and workflows, making the transition smoother. Providing comprehensive training and continuous support can alleviate concerns and build competency in using new technologies [81].

For the public, educational campaigns that highlight the benefits and safety of digital health solutions can help build trust. Transparency in how data are collected, used, and protected is crucial to addressing privacy concerns. Additionally, involving community leaders and trusted healthcare providers in promoting digital health tools can enhance credibility and acceptance [82]. Creating pilot programs and success stories can also demonstrate the effectiveness of digital health solutions, providing tangible evidence of their benefits. Sharing these positive outcomes can help overcome skepticism and encourage wider adoption. By addressing resistance to change through targeted engagement, education, and support, the integration of digital health solutions can be accelerated, leading to improved immunization coverage and more effective health monitoring. Of course, strengthening data protection frameworks is critical for the successful integration of digital health solutions with immunization strategies. As digital health tools handle sensitive personal health information, ensuring the privacy and security of these data is paramount. Robust data protection measures are necessary to address privacy concerns and build public trust [83].

One of the key components of strengthening data protection is the implementation of comprehensive regulations that govern data collection, storage, and usage. These regulations should align with international standards, such as the General Data Protection Regulation (GDPR) in Europe [84] or the Health Insurance Portability and Accountability Act (HIPAA) in the United States [85]. Compliance with these standards ensures that personal health data is handled with the highest level of security and privacy. Another essential aspect is the adoption of advanced security technologies to protect data from breaches and unauthorized access [86]. Encryption, secure access controls, and regular security audits are crucial in safeguarding health information. Implementing multi-factor authentication and ensuring that data are anonymized, when possible, can further enhance security. Transparency in data usage is also vital in building public trust. Individuals should be informed about what data are being collected, how they are being used, and who has access to them. Clear communication about data protection policies and practices can help alleviate fears and misconceptions about digital health tools. Additionally, giving users control over their data, such as the ability to opt-out of certain data collection practices or to request the deletion of their information, can empower them and increase their confidence in digital health solutions. Training for healthcare professionals on data protection best practices is also important. Ensuring that those who handle sensitive information are well-versed in privacy regulations and security protocols can minimize the risk of data breaches and misuse. Moreover, fostering a culture of accountability within organizations that manage health data is essential. Establishing clear lines of responsibility for data protection and implementing regular oversight can ensure that data protection measures are consistently applied and updated as needed.

By focusing on these strategies to strengthen data protection frameworks, it is possible to address privacy concerns effectively and build the necessary public trust in digital health solutions. This trust is crucial for the successful adoption and integration of these technologies, which can significantly enhance immunization coverage and monitoring.

## 5. Conclusions

In conclusion, integrating digital health solutions with immunization strategies holds immense potential for improving immunization coverage and monitoring, especially in the post-COVID-19 era. However, they also come with potential pitfalls that could lead to developments in incorrect or suboptimal directions. Digital systems can be complex and may require training for users. If healthcare providers or administrators are not adequately trained, they might misuse or misinterpret the technology, leading to errors in vaccine delivery or tracking. Moreover, these need to be integrated with existing healthcare infrastructure to be effective. Poor integration can lead to fragmented data and inefficiencies, undermining the overall immunization strategy. To mitigate these risks, it is crucial to approach the integration of digital health solutions with careful planning, stakeholder engagement, and ongoing evaluation. This review has highlighted several key aspects and challenges of this integration, emphasizing the importance of addressing the digital divide, overcoming resistance to change, and ensuring robust data protection. Ensuring equitable access to digital health tools requires significant investments in digital infrastructure, particularly in underserved and rural areas. By improving internet connectivity and providing affordable technology, we can close the gap in access and ensure that more populations benefit from digital health innovations. Educational programs aimed at enhancing digital literacy for both healthcare providers and the general public are crucial. These programs can foster greater acceptance and effective use of digital tools, making the transition to new technologies smoother and more efficient. Overcoming resistance to change among healthcare professionals and the public is another critical aspect. Engaging these stakeholders early in the development and implementation process, providing comprehensive training, and demonstrating the benefits of digital health solutions through pilot programs and success stories can help build trust and acceptance. Clear communication about the advantages of digital tools and how they improve patient care and immunization efforts is essential in this regard. Strengthening data protection frameworks is paramount for building public trust in digital health solutions. Implementing robust data protection measures, complying with international standards, and ensuring transparency in data usage are vital steps. By safeguarding sensitive health information and addressing privacy concerns, we can foster a secure environment where digital health tools are trusted and widely adopted. The integration of digital health solutions with immunization strategies offers a transformative opportunity to enhance immunization coverage and monitoring. By addressing the key challenges and implementing strategic measures, digital health solutions can significantly improve public health outcomes. In the post-COVID-19 era, leveraging digital innovations can lead to more efficient and effective immunization programs, ultimately contributing to better health for all.

## References

[1] Tripathi T. (2023). Advances in vaccines: Revolutionizing disease prevention. Sci. Rep..

[2] Immunization Agenda 2030. https://www.who.int/teams/immunization-vaccines-and-biologicals/strategies/ia2030.

[3] Mascola J.R., Fauci A.S. (2020). Novel vaccine technologies for the 21st century. Nat. Rev. Immunol..

[4] van Riel D., de Wit E. (2020). Next-generation vaccine platforms for COVID-19. Nat. Mater..

[5] Shattock A.J., Johnson H.C., Sim S.Y., Carter A., Lambach P., Hutubessy R.C., Thompson K.M., Badizadegan K., Lambert B., Ferrari M.J. (2024). Contribution of vaccination to improved survival and health: Modelling 50 years of the Expanded Programme on Immunization. Lancet.

[6] Carter A., Msemburi W., Sim S.Y., Gaythorpe K.A., Lambach P., Lindstrand A., Hutubessy R. (2023). Modeling the impact of vaccination for the immunization Agenda 2030: Deaths averted due to vaccination against 14 pathogens in 194 countries from 2021 to 2030. Vaccine.

[7] Progress and Challenges with Achieving Universal Immunization Coverage. https://www.who.int/publications/m/item/progress-and-challenges.

[8] World Health Organization (1979). Global Commission for the Certification of Smallpox Eradication. The Global Eradication of Smallpox: Final Report of the Global Commission for the Certification of Smallpox Eradication..

[9] Polio Eradication Strategy 2022–2026: Delivering on a Promise. https://www.who.int/publications/i/item/9789240031937.

[10] Malaria Vaccine Implementation Programme. https://www.who.int/initiatives/malaria-vaccine-implementation-programme.

[11] One-Dose Human Papillomavirus (HPV) Vaccine Offers Solid Protection against Cervical Cancer. https://www.who.int/news/item/11-04-2022-one-dose-human-papillomavirus-(hpv)-vaccine-offers-solid-protection-against-cervical-cancer.

[12] COVID-19 Vaccines | WHO COVID-19 Dashboard. https://data.who.int/dashboards/covid19/vaccines.

[13] Filip R., Gheorghita Puscaselu R., Anchidin-Norocel L., Dimian M., Savage W.K. (2022). Global challenges to public healthcare systems during the COVID-19 pandemic: A review of pandemic measures and problems. J. Pers. Med..

[14] Pavia G., Spagnuolo R., Quirino A., Marascio N., Giancotti A., Simeone S., Cosco C., Tino E., Carrabetta F., Di Gennaro G. (2023). COVID-19 Vaccine Booster Shot Preserves T Cells Immune Response Based on Interferon-Gamma Release Assay in Inflammatory Bowel Disease (IBD) Patients on Anti-TNF*α* Treatment. Vaccines.

[15] Lazarus J.V., Wyka K., White T.M., Picchio C.A., Gostin L.O., Larson H.J., Rabin K., Ratzan S.C., Kamarulzaman A., El-Mohandes A. (2023). A survey of COVID-19 vaccine acceptance across 23 countries in 2022. Nat. Med..

[16] ECDC COVID-19 Vaccine Tracker. https://vaccinetracker.ecdc.europa.eu/.

[17] WHO/UNICEF Estimates of National Immunization Coverage. https://www.who.int/teams/immunization-vaccines-and-biologicals/immunization-analysis-and-insights/global-monitoring/immunization-coverage/who-unicef-estimates-of-national-immunization-coverage.

[18] Cousins S. (2019). Measles: A global resurgence. Lancet Infect. Dis..

[19] Bigouette J.P. (2023). Update on vaccine-derived poliovirus outbreaks—worldwide, January 2021–December 2022. MMWR. Morb. Mortal. Wkly. Rep..

[20] Shet A., Carr K., Danovaro-Holliday M.C., Sodha S.V., Prosperi C., Wunderlich J., Wonodi C., Reynolds H.W., Mirza I., Gacic-Dobo M. (2022). Impact of the SARS-CoV-2 pandemic on routine immunisation services: Evidence of disruption and recovery from 170 countries and territories. Lancet Glob. Health.

[21] Causey K., Fullman N., Sorensen R.J., Galles N.C., Zheng P., Aravkin A., Danovaro-Holliday M.C., Martinez-Piedra R., Sodha S.V., Velandia-González M.P. (2021). Estimating global and regional disruptions to routine childhood vaccine coverage during the COVID-19 pandemic in 2020: A modelling study. Lancet.

[22] O’Brien K.L., Lemango E. (2023). The big catch-up in immunisation coverage after the COVID-19 pandemic: Progress and challenges to achieving equitable recovery. Lancet.

[23] WHO Immunization Dashboard. https://immunizationdata.who.int/.

[24] Immunization Agenda 2030. https://www.immunizationagenda2030.org/.

[25] Getachew E., Adebeta T., Muzazu S.G., Charlie L., Said B., Tesfahunei H.A., Wanjiru C.L., Acam J., Kajogoo V.D., Solomon S. (2023). Digital health in the era of COVID-19: Reshaping the next generation of healthcare. Front. Public Health.

[26] Sun T., He X., Song X., Shu L., Li Z. (2022). The digital twin in medicine: A key to the future of healthcare?. Front. Med..

[27] Manteghinejad A., Javanmard S.H. (2021). Challenges and opportunities of digital health in a post-COVID19 world. J. Res. Med. Sci..

[28] Budd J., Miller B.S., Manning E.M., Lampos V., Zhuang M., Edelstein M., Rees G., Emery V.C., Stevens M.M., Keegan N. (2020). Digital technologies in the public-health response to COVID-19. Nat. Med..

[29] Odone A., Gianfredi V., Sorbello S., Capraro M., Frascella B., Vigezzi G.P., Signorelli C. (2021). The use of digital technologies to support vaccination programmes in Europe: State of the art and best practices from experts’ interviews. Vaccines.

[30] Hou J., Zhao R., Gronsbell J., Lin Y., Bonzel C.L., Zeng Q., Zhang S., Beaulieu-Jones B.K., Weber G.M., Jemielita T. (2023). Generate Analysis-Ready Data for Real-world Evidence: Tutorial for Harnessing Electronic Health Records With Advanced Informatic Technologies. J. Med. Internet Res..

[31] Kim H.N., Moore K.L., Sanders D.L., Jackson M., Cohen C., Andrews R., Graham C.S. (2024). Implementing Adult Hepatitis B Immunization and Screening Using Electronic Health Records: A Practical Guide. Vaccines.

[32] Volk J.E., Leyden W.A., Lea A.N., Lee C., Donnelly M.C., Krakower D.S., Lee K., Liu V.X., Marcus J.L., Silverberg M.J. (2024). Using Electronic Health Records to Improve HIV Preexposure Prophylaxis Care: A Randomized Trial. JAIDS J. Acquir. Immune Defic. Syndr..

[33] Van Dort B.A., Zheng W.Y., Sundar V., Baysari M.T. (2021). Optimizing clinical decision support alerts in electronic medical records: A systematic review of reported strategies adopted by hospitals. J. Am. Med. Inform. Assoc..

[34] Keshta I., Odeh A. (2021). Security and privacy of electronic health records: Concerns and challenges. Egypt. Inform. J..

[35] Sher M.L., Talley P.C., Yang C.W., Kuo K.M. (2017). Compliance with electronic medical records privacy policy: An empirical investigation of hospital information technology staff. INQUIRY J. Health Care Organ. Provis. Financ..

[36] Basil N.N., Ambe S., Ekhator C., Fonkem E., Nduma B.N., Ekhator C. (2022). Health records database and inherent security concerns: A review of the literature. Cureus.

[37] Woldemariam M.T., Jimma W. (2023). Adoption of electronic health record systems to enhance the quality of healthcare in low-income countries: A systematic review. BMJ Health Care Inform..

[38] Jonnatan L., Seaton C.L., Rush K.L., Li E.P., Hasan K. (2022). Mobile device usage before and during the COVID-19 pandemic among rural and urban adults. Int. J. Environ. Res. Public Health.

[39] Oladepo O., Dipeolu I.O., Oladunni O. (2021). Outcome of reminder text messages intervention on completion of routine immunization in rural areas, Nigeria. Health Promot. Int..

[40] Uddin M.J., Shamsuzzaman M., Horng L., Labrique A., Vasudevan L., Zeller K., Chowdhury M., Larson C.P., Bishai D., Alam N. (2016). Use of mobile phones for improving vaccination coverage among children living in rural hard-to-reach areas and urban streets of Bangladesh. Vaccine.

[41] Gilano G., Sako S., Molla B., Dekker A., Fijten R. (2024). The effect of mHealth on childhood vaccination in Africa: A systematic review and meta-analysis. PLoS ONE.

[42] Gibson D.G., Ochieng B., Kagucia E.W., Were J., Hayford K., Moulton L.H., Levine O.S., Odhiambo F., O’Brien K.L., Feikin D.R. (2017). Mobile phone-delivered reminders and incentives to improve childhood immunisation coverage and timeliness in Kenya (M-SIMU): A cluster randomised controlled trial. Lancet Glob. Health.

[43] Shaw R., Stroo M., Fiander C., McMillan K. (2020). Selecting mobile health technologies for electronic health record integration: Case study. J. Med. Internet Res..

[44] Katusiime J., Tumuhimbise W., Rwambuka Mugyenyi G., Kobutungi P., Mugaba A., Zender R., Pinkwart N., Musiimenta A. (2022). The role of mobile health technologies in promoting COVID-19 prevention: A narrative review of intervention effectiveness and adoption. Digit. Health.

[45] Rajput A.R., Masood I., Tabassam A., Aslam M.S., ShaoYu Z., Rajput M.A. (2023). Patient’s data privacy and security in mHealth applications: A Charles proxy-based recommendation. Soft Comput..

[46] Stoumpos A.I., Kitsios F., Talias M.A. (2023). Digital transformation in healthcare: Technology acceptance and its applications. Int. J. Environ. Res. Public Health.

[47] Hatef E., Wilson R.F., Zhang A., Hannum S.M., Kharrazi H., Davis S.A., Foroughmand I., Weiner J.P., Robinson K.A. (2024). Effectiveness of telehealth versus in-person care during the COVID-19 pandemic: A systematic review. NPJ Digit. Med..

[48] Doraiswamy S., Abraham A., Mamtani R., Cheema S. (2020). Use of telehealth during the COVID-19 pandemic: Scoping review. J. Med. Internet Res..

[49] Singh J., Albertson A., Sillerud B. (2022). Telemedicine during COVID-19 crisis and in post-pandemic/post-vaccine world—historical overview, current utilization, and innovative practices to increase utilization. Healthcare.

[50] Li C., Borycki E.M., Kushniruk A.W. (2021). Connecting the world of healthcare virtually: A scoping review on virtual care delivery. Healthcare.

[51] Ye J., He L., Beestrum M. (2023). Implications for implementation and adoption of telehealth in developing countries: A systematic review of China’s practices and experiences. NPJ Digit. Med..

[52] Haleem A., Javaid M., Singh R.P., Suman R. (2021). Telemedicine for healthcare: Capabilities, features, barriers, and applications. Sens. Int..

[53] Tosta S., Moreno K., Schuab G., Fonseca V., Segovia F.M.C., Kashima S., Elias M.C., Sampaio S.C., Ciccozzi M., Alcantara L.C.J. (2023). Global SARS-CoV-2 genomic surveillance: What we have learned (so far). Infect. Genet. Evol..

[54] WHO Immunization Data Portal—Global. http://immunizationdata.who.int.

[55] Pavia G., Quirino A., Marascio N., Veneziano C., Longhini F., Bruni A., Garofalo E., Pantanella M., Manno M., Gigliotti S. (2024). Persistence of SARS-CoV-2 infection and viral intra-and inter-host evolution in COVID-19 hospitalized patients. J. Med. Virol..

[56] De Marco C., Veneziano C., Massacci A., Pallocca M., Marascio N., Quirino A., Barreca G.S., Giancotti A., Gallo L., Lamberti A.G. (2022). Dynamics of viral infection and evolution of SARS-CoV-2 variants in the Calabria area of Southern Italy. Front. Microbiol..

[57] Quality and Use of Global Immunization and Surveillance. https://www.who.int/groups/strategic-advisory-group-of-experts-on-immunization/working-groups/quality-and-use-of-global-immunization-and-surveillance-data.

[58] Buttery J.P., Clothier H. (2022). Information systems for vaccine safety surveillance. Hum. Vaccines Immunother..

[59] Xiang D., Cai W. (2021). Privacy protection and secondary use of health data: Strategies and methods. BioMed Res. Int..

[60] McGraw D., Mandl K.D. (2021). Privacy protections to encourage use of health-relevant digital data in a learning health system. NPJ Digit. Med..

[61] Bravi B. (2024). Development and use of machine learning algorithms in vaccine target selection. NPJ Vaccines.

[62] Sharma A., Virmani T., Pathak V., Sharma A., Pathak K., Kumar G., Pathak D. (2022). Artificial intelligence-based data-driven strategy to accelerate research, development, and clinical trials of COVID vaccine. BioMed Res. Int..

[63] Lian A.T., Du J., Tang L. (2022). Using a machine learning approach to monitor COVID-19 vaccine adverse events (VAE) from twitter data. Vaccines.

[64] Larson H.J., Lin L. (2024). Generative artificial intelligence can have a role in combating vaccine hesitancy. BMJ.

[65] Gerke S., Minssen T., Cohen G. (2020). Ethical and legal challenges of artificial intelligence-driven healthcare. Artificial Intelligence in Healthcare.

[66] Nissar G., Khan R.A., Mushtaq S., Lone S.A., Moon A.H. (2024). IoT in healthcare: A review of services, applications, key technologies, security concerns, and emerging trends. Multimed. Tools Appl..

[67] Kashani M.H., Madanipour M., Nikravan M., Asghari P., Mahdipour E. (2021). A systematic review of IoT in healthcare: Applications, techniques, and trends. J. Netw. Comput. Appl..

[68] Hu H., Xu J., Liu M., Lim M.K. (2023). Vaccine supply chain management: An intelligent system utilizing blockchain, IoT and machine learning. J. Bus. Res..

[69] Jiang S., Jia S., Guo H. (2024). Internet of Things (IoT)-enabled framework for a sustainable Vaccine cold chain management system. Heliyon.

[70] Ali Y., Khan H.U. (2023). IoT platforms assessment methodology for COVID-19 vaccine logistics and transportation: A multi-methods decision making model. Sci. Rep..

[71] Sadek I., Codjo J., Rehman S.U., Abdulrazak B. (2022). Security and privacy in the Internet of Things healthcare systems: Toward a robust solution in real-life deployment. Comput. Methods Programs Biomed. Update.

[72] Landi I., Glicksberg B.S., Lee H.C., Cherng S., Landi G., Danieletto M., Dudley J.T., Furlanello C., Miotto R. (2020). Deep representation learning of electronic health records to unlock patient stratification at scale. NPJ Digit. Med..

[73] Siddiqi D.A., Abdullah S., Dharma V.K., Shah M.T., Akhter M.A., Habib A., Khan A.J., Chandir S. (2021). Using a low-cost, real-time electronic immunization registry in Pakistan to demonstrate utility of data for immunization programs and evidence-based decision making to achieve SDG-3: Insights from analysis of big data on vaccines. Int. J. Med. Inform..

[74] Mallah N., Pardo-Seco J., Rivero-Calle I., Zhu-Huang O., Fernández Prada M., Reynen-de Kat C., Benes O., Mosina L., Sankar-Datta S., Aleksinskaya O. (2024). COVID-19 InfoVaccines: A WHO-supported educational project to promote COVID-19 vaccination information among professionals and the general population. Hum. Vaccines Immunother..

[75] Demsash A.W., Chereka A.A., Walle A.D., Kassie S.Y., Bekele F., Bekana T. (2023). Machine learning algorithms’ application to predict childhood vaccination among children aged 12–23 months in Ethiopia: Evidence 2016 Ethiopian Demographic and Health Survey dataset. PLoS ONE.

[76] Li F. (2022). Disconnected in a pandemic: COVID-19 outcomes and the digital divide in the United States. Health Place.

[77] Borges do Nascimento I.J., Abdulazeem H., Vasanthan L.T., Martinez E.Z., Zucoloto M.L., Østengaard L., Azzopardi-Muscat N., Zapata T., Novillo-Ortiz D. (2023). Barriers and facilitators to utilizing digital health technologies by healthcare professionals. NPJ Digit. Med..

[78] Digital Health Literacy Key to Overcoming Barriers for Health Workers, WHO Study Says. https://www.who.int/azerbaijan/news/item/18-09-2023-digital-health-literacy-key-to-overcoming-barriers-for-health-workers-who-study-says.

[79] Tozzi A.E., Gesualdo F., D’Ambrosio A., Pandolfi E., Agricola E., Lopalco P. (2016). Can digital tools be used for improving immunization programs?. Front. Public Health.

[80] Alscher A., Schnellbächer B., Wissing C. (2023). Adoption of Digital Vaccination Services: It Is the Click Flow, Not the Value—An Empirical Analysis of the Vaccination Management of the COVID-19 Pandemic in Germany. Vaccines.

[81] Guo C., Ashrafian H., Ghafur S., Fontana G., Gardner C., Prime M. (2020). Challenges for the evaluation of digital health solutions—A call for innovative evidence generation approaches. NPJ Digit. Med..

[82] Patient Safety Network (PSNet) Digital Health Literacy. https://psnet.ahrq.gov/primer/digital-health-literacy.

[83] Medical Device Network Cybersecurity and Data Protection Impact on Digital Health. https://www.medicaldevice-network.com/analyst-comment/cybersecurity-data-protection-digital-health/.

[84] General Data Protection Regulation (GDPR). https://gdpr-info.eu/.

[85] Health Insurance Portability and Accountability Act (HIPAA) & Health Information Technology for Economic and Clinical Health (HITECH) Act. https://learn.microsoft.com/it-it/compliance/regulatory/offering-hipaa-hitech.

[86] Fortinet What Is Data Security?. https://www.fortinet.com/resources/cyberglossary/data-security.

